# How to Control the Microfluidic Flow and Separate the Magnetic and Non-Magnetic Particles in the Runner of a Disc

**DOI:** 10.3390/mi12111335

**Published:** 2021-10-30

**Authors:** Yao-Tsung Lin, Chien-Sheng Huang, Shi-Chang Tseng

**Affiliations:** 1 Department of Mechanical Engineering, Chien Hsin University of Science and Technology, Zhongli District, Taoyuan 320312, Taiwan; lcd_lin@yahoo.com.tw; 2 Department of Electronic Engineering, National Yunlin University of Science and Technology, Douliu, Yunlin 64002, Taiwan; 3 Department of Mechanical Engineering, National Yunlin University of Science and Technology, Douliu, Yunlin 64002, Taiwan; tsengsc8@gmail.com

**Keywords:** magnetic-activated, disc, microfluidic, stomach-shape, rectangular-shape, spin

## Abstract

Biochips play an important role in both medical and food industry safety testing. Moreover, magnetic activated cell sorting is a well-established technology for biochip development. However, biochips need to be manufactured by precision instruments, resulting in the high cost of biochips. Therefore, this study used magnetic-activation and mechanics theories to create a novel disc that could manipulate the microfluidic flow, mixing, reaction, and separation on the runner of the disc. The goal of the research was to apply in the field of biomedical detection systems to reduce the cost of biochips and simplify the operation process. The simulation and experimental investigation showed that the pattern of the reaction chamber was stomach-shaped and the reservoir chamber was rectangular-shaped on the disc. The microfluid could be controlled to flow to the reaction chamber from the buffer and sample chamber when the disc spun at 175~200 rpm within three minutes. This was defined as the first setting mode. The microfluid could then be controlled to flow to the reservoir chamber from the reaction chamber when the disc spun at 225 rpm within five to ten minutes. This was defined as the second setting mode. This verified that the pattern design of the disc was optimized for control of the microfluid flow, mixing, reaction, and separation in the runner of the disc by different setting modes.

## 1. Introduction

Biochips have the advantages of providing faster analysis with fewer samples and detect the disease in the early stage. In addition, biochips can be designed with disposal and lower production costs. Disposal biochips can reduce the risk of infections for medical examiners during sample testing. It has led to an increase in research and applications in pathological examinations in recent years [1,2,3,4].

Common methods of cancer diagnosis include biopsies, image examinations, and blood tests. Biopsies are often used for the most accurate determination of tumor characteristics; however, biopsies are invasive and painful procedures that may result in infections. Image examinations, ultrasounds, and X-ray findings are limited by resolution and scale, making them poor at discovering small tumor cells early in their development. Therefore, it would be highly beneficial if medical practitioners had a new means to make quick and accurate diagnoses. The detection sensitivity of CTC is lower, but it can detect multiple cancer genes from the DNA in blood samples [5]. This is used microfluidic devices to sorting circulating tumor cells. The United States Food and Drug Administration (FDA) has confirmed that CTC detection technology is among the best methods and most objective after a long period of data collection. It is therefore advisable for medical practitioners to adopt this technology during pathological examinations [6].

Cell sorting methods can be divided into chromatography membrane filtration, dielectrophoresis (DEP), and flow cytometry [7]. The sensitivity of chromatography membrane filtration and dielectrophoresis is lower than the detection method of flow cytometry. Flow cytometry combined with magnetic beads has the advantage of being a simple device that requires few samples and that has a shorter test time and a lower cost. Thus, it is well suited for the examination of rare cells or virus detection [8,9]. MACS is a representative principle that uses magnetic beads and magnetic field assisted cell sorting [10]. However, bulk separators rely on irregular and dense ferromagnetic matrices that can cause variances in separation purity and recovery. In addition, there also exists the issue of a higher cost on the part of cell separation columns, which makes it too difficult to commercialize.

Microfluidic flow in the disc is one of the methods used for cell sorting. This process has the advantages of low consumption of samples and reagents, high sensitivity, a short analysis time, and a low cost. Rokon et al. and Ehsan Mahmodi et al. used the platform to perform blood sample sorting [11]; however, their disc was assembled using multi-layer platforms and a double chamber design [12,13]. As the boundary layer of the microfluidic flow, the double chamber design of the disc affected the detection accuracy [13,14]. Therefore, this research used a design, simulation, and an experimental investigation to study how to control the microfluidic flow to the expected chamber by centrifugal force, improve the detection efficiency and reduce the cost of the detected substrate.

## 2. Research Procedure and Methods

This research used magnetic activated technology, Newton’s laws of motion, and the law of conservation of energy to create the disc. The disc had the function of controlling the microfluidic flow and separating the magnetic and non-magnetic particles in the runner of the disc. Control of the microfluidic flow consisted of two stages. The first stage was to control the microfluidic flow to the reaction chamber from the buffer and sample chamber fully with the disc spinning ω_1_. The second stage was to control the microfluidic flow to the reservoir chamber from the reaction chamber with the disc spinning ω_2_.

### 2.1. Design the Pattern of the Disc

**Figure 1:**
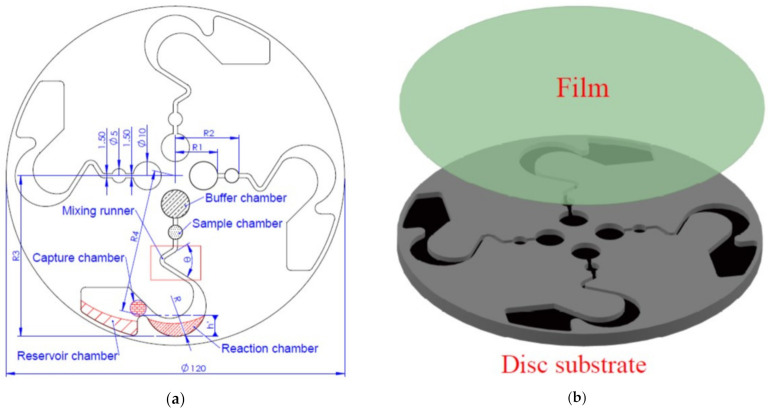
Design of the disc pattern. (**a**) Disc pattern design and labelled chamber locations. (**b**) Disc is assembly by film and disc substrate.

The more calibrated samples in the runner of the disc, the large number of isolated samples will be obtained. This helps to improve the accuracy of detection [15]. Therefore, the pattern of the disc was designed as shown in Figure 1a. The disc was divided into six areas: (i) a buffer chamber; (ii) a sample chamber; (iii) a mixing runner; (iv) a reaction chamber; (v) a capture chamber; and (vi) a reservoir chamber. Each chamber was connected with the runner and adopted a single chamber design. The disc was assembled by film and disc substrate, as shown in Figure 1b.

In order to increase the effect of mixing, reaction ability, and flow to the reservoir chamber when the microfluidics flows in the pattern of the disc by the centrifugal force [16,17]. The mixing runner was designed with a curved shape, the reaction chamber was designed with a stomach-like shape, and the reservoir chamber was designed with a rectangle-like shape. The mixing runner’s angle was θ, and h′ was the distance between the reaction chamber and reservoir chamber, as shown in Figure 1a.

### 2.2. Theory

The theory is according to mechanics theories to create the patterns of the disc. The first stage of the disc spinning was according to Newton’s laws of motion and used to control the microfluidic flow. The second stage was according to the law of conservation of energy [18,19].

Newton’s laws of motion:

Centrifugal force F_Ce_ is described by Equation (1):

F_Ce_ = *m·ω*_1_^2^·*r* (1)

and static friction force F_Sf_ is described by Equation (2):

F_Sf_ = *μ_s_*·*N* = *μ_s_*·*m*·*g* (2)

where *m* is the mass of a particle, *g* is the acceleration due to gravity; and *μ_s_* is the static friction coefficient.

The microfluids or particles will flow to the reaction chamber from the buffer and sample chamber when F_Ce_ > F_Sf_.

The law of conservation of energy:

Kinetic energy E_k_ is described by Equation (3):

E_k_ = 1/2·*m*·v^2^ (3)

and potential energy E_p_ is described by Equation (4):

E_p_ = *m·g*·h′ (4)

The microfluids or particles will flow to the reservoir chamber from the reaction chamber when E_k_ > E_p_.

### 2.3. Simulation Method

**Figure 2:**
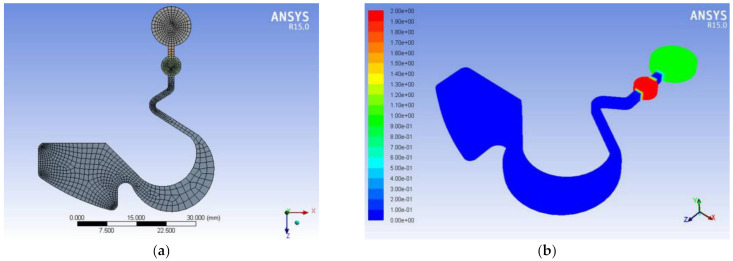
Grid cutting and boundary conditions in the pattern of the disc. (**a**) Mesh cutting of the disc runner structure. (**b**) Boundary condition set on the buffer and sample chamber.

Ansys Fluent R15.0 uses the hexahedral finite element mesh and numerical calculations to analyze the flow field. Compared with real flow experiments, it is possible to investigate to determine the optimal design patterns for the disc by CAE software. It is also possible to correct and avoid potential design errors by computing the outcome of the microfluidic flow [20,21]. Therefore, this study used Ansys Fluent R15.0 to create the optimal pattern design of the disc. Figure 2a shows the hexahedral mesh finite element according to the pattern design of Figure 1. Figure 2b and Table 1 shows the setting of the boundary conditions, in which the green circle is a buffer liquid, the red circle is the sample liquid and the blue area is the runner on the disc.

### 2.4. Experimental Method

**Figure 3:**
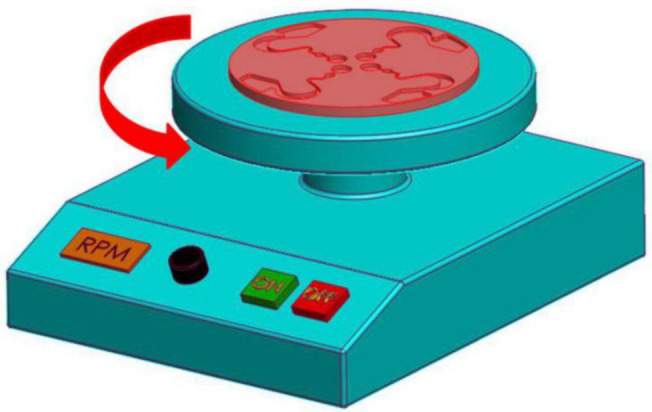
Schematic of the disc design on the spinning table and setting modes of the centrifuge.

Upon determining the optimal pattern design, a CNC machine was used to manufacture the patterns of the disc [22]. The disc was then placed in the rotating table, as shown in Figure 3, to test the flow and separation functions of the magnetic and non-magnetic particles.

Both the simulation and experimental sections had two stages in each cycle. The first stage studied how to control the microfluidic or particle flow to the reaction chamber from the buffer or sample chamber. Then, the second stage was control of the microfluidic or particle flow to the reservoir chamber from the reaction chamber, which was done by disc rotation. At this point, the magnetic particles were captured at the capture zone and non-magnetic particles how flowed to the reservoir chamber. As a result, it was possible to see the separation of the magnetic and non-magnetic particles.

## 3. Simulation and Experimental Result

### 3.1. Optimal Pattern Design and Rotating Mode of the Centrifugal Disc by Simulation

#### 3.1.1. Optimal Mixing Runner Design

**Figure 4:**
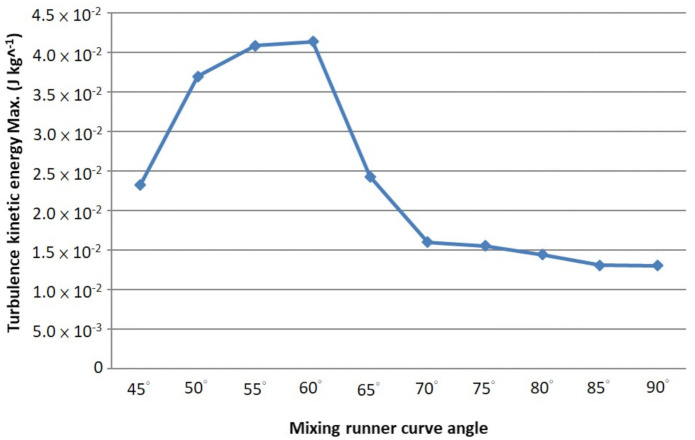
The transient maximum turbulence kinetic energy of the microfluidic in the mixing runner area during the first stage of rotation in the disc.

The mixing runner’s curve angle was θ in the disc, as shown in Figure 1. Differences in the disturbance of the microfluidic were difficult to observe or analyze when the microfluidic flow was in the mixing runner of the spanned disc. As flow lasts too short, they are laminar flow [21]. Therefore, several different curve angles were designed for mixing the runners to simulate whether they would have different transient disturbances with the microfluidic flow into the mixing runner. The analysis result is displayed in Figure 4. Different mixing runner designs showed different disturbances in the kinetic energy values. The maximum value occurred when the mixing runner’s curve angle was designed between 55° and 60°. Therefore, we accordant the 60° of the mixing runner to set the other boundary conditions and simulation.

#### 3.1.2. Optimal Rotating Mode Setting of the Disc by Simulation

The centrifugal disc was rotated at two different speed modes. The microfluidics flowed to the reaction chamber from the buffer and sample chamber in the first mode, and then the microfluidics flowed into the reservoir chamber under different rotating modes. The first mode was according to Newton’s laws of motion. According to the value of the theoretical calculation, the disc of the first rotating speed was set 100 rpm and 200 rpm individually. The second rotating mode of the speed was set at 250 rpm. The boundary value setting of the simulation is illustrated in Table 2.

**Figure 5:**
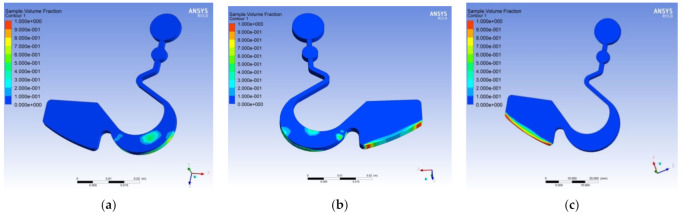
Optimal pattern design and simulation results of the disc. (**a**) The simulation result of the microfluidic flow after the disc spun at the first setting mode at a rotating speed of 100 rpm. (**b**) The simulation result of the microfluidic flow after the disc spun at the first setting mode at a rotating speed of 200 rpm. (**c**) The simulation result of the microfluidic flow after the disc spun at the second setting mode at a rotating speed of 250 rpm.

Figure 5 shows the simulation result according to the finite element mesh of the disc runner structure shown in Figure 2. It was observed that the sample fluid remained fully in the reaction chamber. It did not have the phenomenon of overflowing to the reservoir chamber when the disc rotating speed was set at 100 rpm, as shown in Figure 5a. The sample fluid would overflow to the reservoir chamber from the reaction chamber when the disc rotating speed was increased to 200 rpm as shown in Figure 5b. The microfluidic flow to the reservoir chamber from the reaction chamber completely when the disc rotated at 250 rpm, as shown in Figure 5c.

Following the above design and the CAE simulation results, the optimal boundary conditions of the first stage mode, including the pattern of the reaction zone, was designed as a stomach shape, the reservoir zone was designed as a rectangular shape, and the disc rotating speed was set 100 rpm. There will be no overflow to other areas or produce a backflow phenomenon at the first stage’s setting mode. It appeared possible that the optimal design of the disc would be able to govern the microfluidic flow to the reaction and reservoir chambers by judicious control of the spin speed. To test this extended theory, further experiments were designed and conducted.

### 3.2. The Experimental of Flowing and Sorting in the Centrifugal Disc for Magnetic and Non-Magnetic Particles

**Figure 6:**
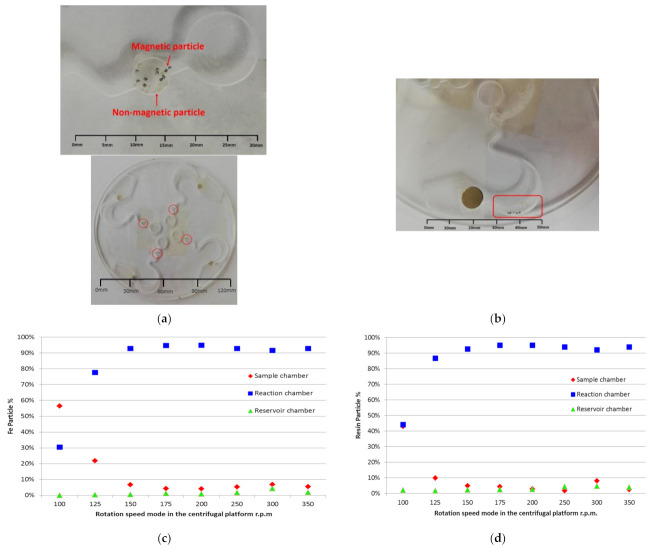
Ratio of magnetic and non-magnetic particles flowing to the reaction chamber under different spinning speeds of the disc in the first setting mode. (**a**) Magnetic and non-magnetic particles in the sample ready for testing. (**b**) Magnetic and non-magnetic particles flowing to the reaction chamber from the sample chamber of the disc. (**c**) Magnetic particles (Fe) flowing to the reaction chamber from the sample chamber of the disc. (**d**) Non-magnetic particles (resin) flowing to the reaction chamber from the sample chamber of the disc.

This study used a transparent plastic plate made of PMMA material for the centrifugal disc. The disc’s pattern was according to that shown in Figure 1. Figure 6a is the experimental disc by CNC machine manufacturing [23]. Ten pieces of magnetic and non-magnetic particles were placed in the sample chamber individually, as shown in Figure 6a. The material of the magnetic particles was Fe, and the material of the non-magnetic particles was a resin. The average weight of the Fe was about 4.25 × 10^−4^ g and the diameter was ψ0.3 ± 0.05 mm. The average weight of the resin was about 1.32 × 10^−4^ g and the diameter was ψ0.5 ± 0.1 mm. A magnet was placed in the capture chamber, as shown in Figure 6b. The magnet size was ψ10 × 2.0 mm and the average force was about 800 Gauss.

Different rotating speeds were set in the first mode of the disc. The rotating speed range was between 100 rpm and 350 rpm, and the rotating time was three minutes. Figure 6b–d show the experimental results. It was found that over 92% of the particles stayed in the reaction chamber when the rotating speed was set in the range of 175 rpm to 200 rpm, as shown in Figure 6a,b. The theoretical calculation value was 149–168 rpm according to Formulas (1) and (2). The experimental results confirmed that the rotating speed of the experimental value was near the theoretical calculation value.

**Figure 7:**
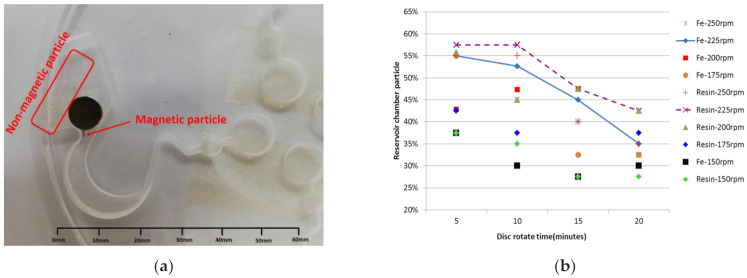
Magnetic and non-magnetic particles flowing to the reservoir chamber from the reaction chamber in the second setting. (**a**) Magnetic particles adsorbed in the capture chamber and non-magnetic particles flowing to the reservoir chamber. (**b**) Ratio of the magnetic particles (Fe) adsorbed in the capture chamber.

The disc spin in the second mode achieved the function of separation of the magnetic and non-magnetic particles according to the law of conservation of energy. Figure 7a shows that the magnetic particles (Fe) adsorbed in the capture chamber and non-magnetic particles (resin) flowed to the reservoir chamber after the disc spun in the second mode. As shown in Figure 7b, the experimental data demonstrated that the disc spun after the second mode. It was found that over 50% of the magnetic particles (Fe) adsorbed in the capture chamber and non-magnetic particles (resin) flowed to the reservoir chamber when the spinning speed was set at 225 rpm and the spinning time was set at 5–10 min on the disc.

Summarizing the experimental data, the optimal condition of the first setting mode was disc spinning at 175–200 rpm for under three minutes. The optimal condition of the second setting mode was disc spinning at 225 rpm under 5–10 min, as shown in Table 3. Total processing was finished within 20 min.

## 4. Conclusions and Future Work

Both CAE analysis and experimented. One experiment which has been done in this research is that microfluidics can be controlled in the runner of the disc through the rotating centrifugal force. The other is that the disc designed in this work had the function of separating magnetic and non-magnetic particles through the use of different setting modes and a magnet. Total processing was finished within 20 min. Therefore, the surface finish and dimensions of the runner in the disc could be optimized through CAE simulation and experimental verification. Future work will study the issues of cell sorting, the magnet polarity adsorption phenomenon, and the individual extraction of magnetic and non-magnetic particles.

## Figures and Tables

**Table 1:** Illustration of the volume design for the buffer liquid and sample liquid.

Items	Volume
Buffer liquid	157.0 μL
Sample liquid	39.0 μL

**Table 2:** Illustration of the boundary value setting with Ansys Fluent analysis.

Items	Value
Buffer flow density/viscosity.	998.2 kg/cm^3^/0.001003 kg/m∙s
Sample flow density/viscosity.	1060 kg/cm^3^/0.003 kg/m∙s
Disc rotating angular velocity at the first setting mode	Constant 100 rpm, 200 rpm/3 min
Disc rotating angular velocity at the second setting mode	Constant 250 rpm/5 min

**Table 3:** Optimal operator conditions for controlling the magnetic and non-magnetic particles flowing and separating in the runner of the disc.

Items	Value
Buffer chamber scale	ψ10 mm × h 2.0 mm
Sample chamber scale	ψ5 mm × h 2.0 mm
Mixing runner cure angle *θ*	60°
The magnet scale	ψ10 × 2.0 mm/800 Gauss
Rotating speed in the first mode of the disc	Constant 175 rpm to 200 rpm/3 min.
Rotating speed in the second mode of the disc	Constant 225 rpm/5~10 min.
